# Ganglion cysts of the cruciate ligaments: a series of 31 cases and review of the literature

**DOI:** 10.1186/1471-2474-13-137

**Published:** 2012-08-03

**Authors:** Yongtao Mao, Qirong Dong, Yi Wang

**Affiliations:** 1 The Orthopaedic Department of the Second Affiliated Hospital of Soochow University, No. 1055 of San Xiang Road, Suzhou 215004, China

**Keywords:** Ganglion cyst, Cruciate ligament, MR imaging, Arthroscopic management

## Abstract

**Background:**

A case series for ganglion cyst of the cruciate ligament with MRI findings, clinical presentation, and management options along with review of literature is presented.

**Methods:**

Of 8663 consecutive patients referred for knee MR imaging, 31 were diagnosed with ganglion cysts of the cruciate ligaments, including 21 men and 10 women of ages 12 to 73 years (mean: 37). A review of charts revealed that knee pain was the chief complaint in all cases. Arthroscopic debridement of ganglion cyst was performed in 11 patients.

**Results:**

MRI proved to be a valuable tool in diagnosing and deciding management of these cases. All 11 patients who underwent arthroscopic treatment were symptom-free on a minimum follow-of one year.

**Conclusion:**

Cyst formation associated with cruciate ligament of the knee is an infrequent cause of knee pain. MR imaging was important in confirming the cyst lesions and provided useful information prior to arthroscopy. Arthroscopic debridement of ganglion cyst produced excellent outcome without recurrence. This study describes the pertinent MRI and intraoperative findings of ganglion cyst.

## Background

Ganglion is a cystic swelling that usually arises close to the tendons or joints. Most often it is encountered over the dorsum of the hand, but it can occur in any part of the body. Ganglion cyst of the cruciate ligament is a rare condition that is seen infrequently in clinical setting, first described in 1924 by Caan in a cadaveric specimen [1]. Most of ligament cysts (75.4%) in the knee joint are known to be located in the anterior cruciate ligament [1]. The exact pathogenesis of the condition is still unknown, although several theories of cyst development have been proposed, such as mucinous degeneration of connective tissue, synovial herniation, congenitally displaced synovial tissue, and trauma leading to degeneration and cyst formation [1-4]. The majority of the patients present with knee pain, although other mechanical symptoms, like locking, snapping, and giving away, are present occasionally. Given the fact that knee pain can be caused by a variety of conditions, diagnosis of ganglion cyst can rarely be made on clinical grounds alone, and frequently it is the MRI findings that lead to the diagnosis. The existing literature on this condition is composed of isolated case reports or small case series. Our study is comparatively a large study of 31 patients, by which we describe various findings on MRI and clinical presentation and discuss the appropriate arthroscopic management and outcomes.

## Methods

The Ethics Committee at our hospital approved the research project. We reviewed the medical records of 8663 consecutive patients referred for knee MR imaging between October 2002 and January 2010. Using the key words of cruciate ligament and cyst, we found 31 patients with ganglion cysts of the cruciate ligaments in the mini-PACS system (Beijing Silver Medical Information Technical Co. Ltd.). All authors in image station verified the MR images. There were 21 men and 10 women, ranging in age from 12 to 73 years (mean: 37). Knee pain, especially with extreme flexion or extension, similar to the symptoms of meniscus tears, was the main complaint. The physical findings were not pathognomonic in any of the patients, and MRI scan was performed in all the cases. All patients had been scanned on a 0.2-T MR unit (Artoscan, Esaote) with a dedicated knee coil. The imaging protocol included spin-echo T1-weighted (TR/TE 560/24 ms), T2-weighted (2060/80 ms) in the coronal, sagittal, and axial planes. The section thickness was 4.5 mm with a gap of 0.4 mm.

Arthroscopic debridement of ganglion cyst was performed in 11 patients, including eight men and three women with a mean age of 35.3 years (range: 23 to 51). The postoperative protocol varied according to the other possible findings and procedures performed in addition to the cyst removal. The patients were followed postoperatively one year. The other patients refused the surgical intervention despite appropriate counseling and were, thus, managed conservatively.

## Results

All patients had the symptoms of knee pain with extreme flexion or extension. No patient had a clear history of injury. Arthroscopic resection was suggested for all the MRI-confirmed cases. Eleven of the diagnosed cases underwent surgical intervention in the form of arthroscopic debridement.

**Figure 1:**
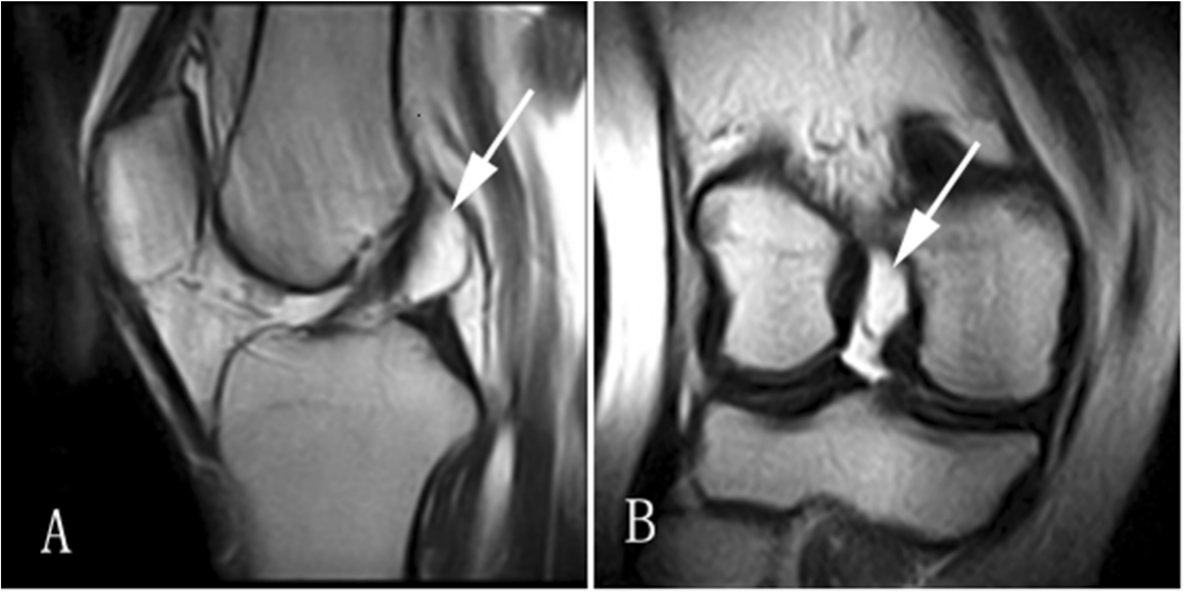
** Cyst of the ACL on T2-weighted image.** (**A**) The ACL was twisted, due to presence of the cyst in the sagittal plane. (**B**) The cysts of the ACL in the coronal plane.

**Figure 2:**
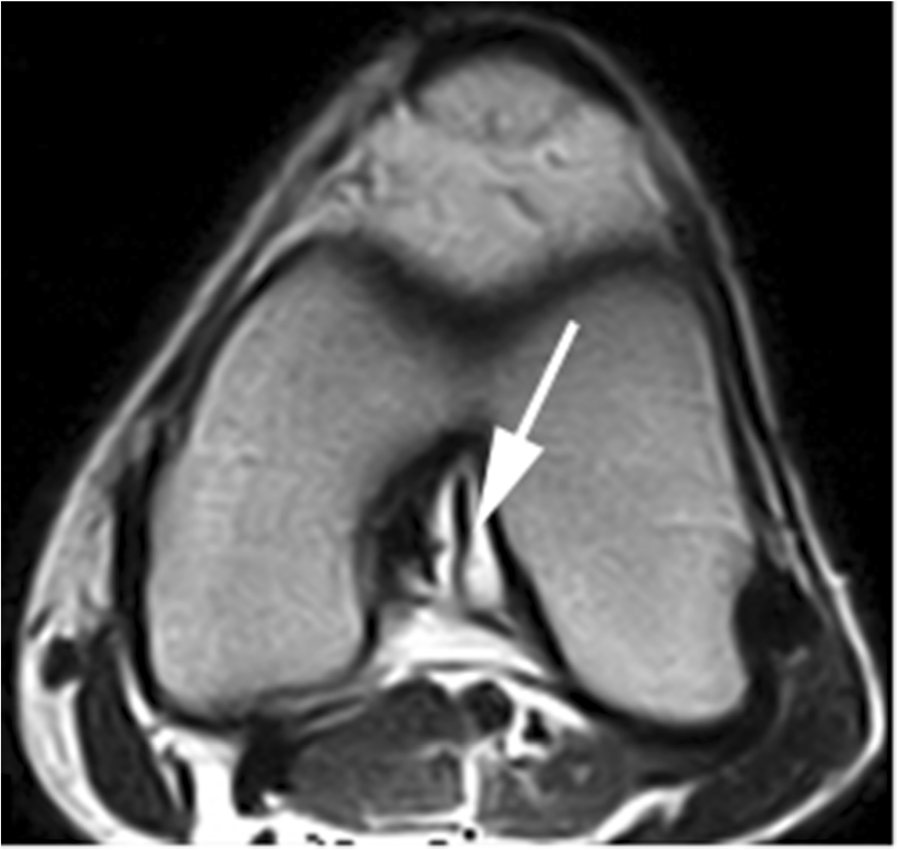
The cyst encompassing the ACL spread between the ACL and PCL on T2-weighted images.

**Figure 3:**
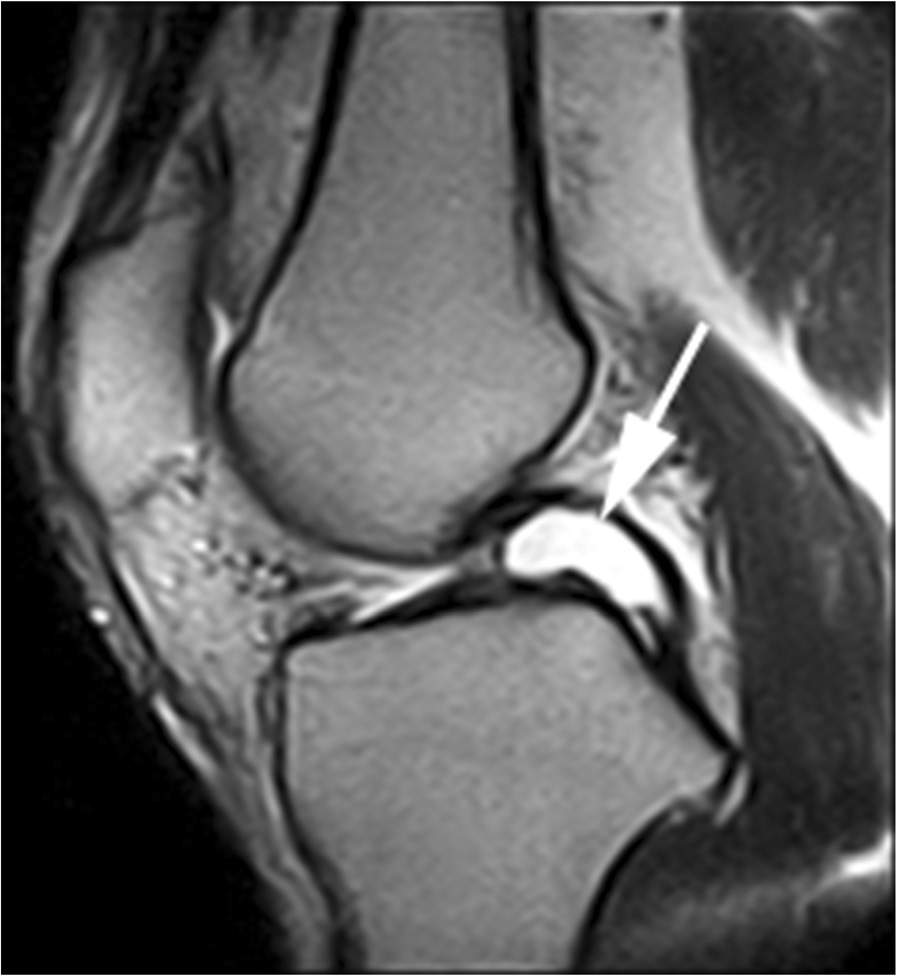
Cyst of the PCL in the sagittal plane on T2-weighted images.

MR imaging: All cysts were isointense to fluid, depicting low signal intensity on T1-weighted images and high signal intensity on T2-weighted images. Twenty-eight cysts were found in the anterior cruciate ligaments (ACL) and three in the posterior cruciate ligaments (PCL). Most of the cysts were ellipse- or round-shaped with a clear boundary. A few of the cysts appeared lobulated or contained internal septa. The cysts of the ACL were firmly adhered to the ligament. When the cyst was large enough, the ligament was seen to be pushed to the lateral side, resulting in a twist-shaped ACL as seen on arthroscopy or interpreted on MRI (Figure 1). The cyst wall margin extended from the ACL to the PCL in four cases, suggesting that the origin of the cyst could be the space between the ACL and PCL (Figure 2). The cysts of PCL were mainly below the ligament (Figure 3). Degenerative changes in knee joint were observed in six cases.

**Figure 4:**
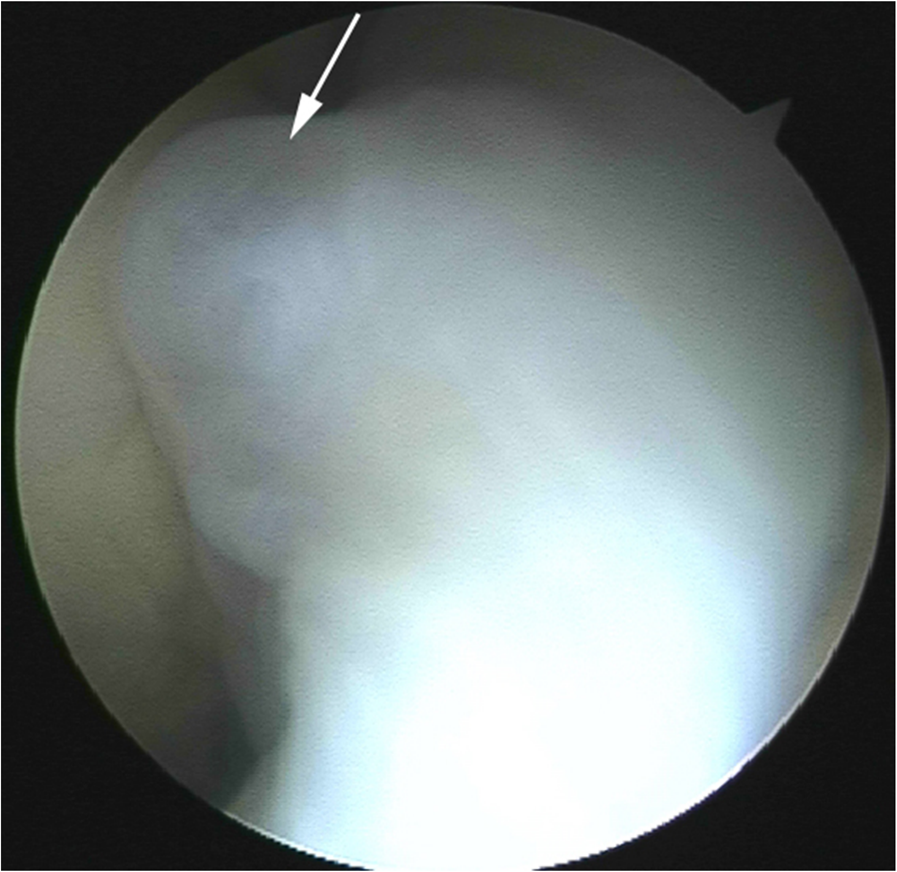
An ellipse-shaped cyst as seen on arthroscopy.

Arthroscopic resection: Arthroscopic treatment was performed in 11 patients. The location of the cysts correlated to MR findings in that the shape of cysts was mainly round or elliptical. Lobulated structures and internal septa were distinguishable in some cysts (Figure 4). During arthroscopy, all cysts were excised using a motorized shaver. Viscous fluid with a slightly yellow color exuded when the cysts were being excised. Tissues of synovial membrane, collagen, and fascia were discovered in eight cysts upon histological examination after operation.

Follow-up: One patient suffered from acute hematoma in the knee joint after the operation and recovered after an additional surgery of knee arthrotomy and drainage. All eleven patients who had arthroscopic treatment were symptom-free at follow-up after one year.

## Discussion

In this study, we found that MR imagines are valuable for the detection of these uncommon cysts, and arthroscopic management is effective. Thirty one cysts were found by MR examinations, giving an incidence of 0.36%. The incidence of patients undergoing surgical intervention for management of this condition was 0.88% with arthroscopic resection of the cysts being performed in 11 patients of 1253 cases that were screened and formed the part of this study. In a similar large series on ganglion cysts reported so far, Sarimo et al. found nine of 2400 knees demonstrated a cyst associated with a cruciate ligament upon arthroscopic examinations [5]. Fifteen cases of ganglion cysts (1.10% of all 1364 arthroscopies) relating to the ACL were reported by Parish in another study [6].

The youngest patient in the medical literature was a child of two years old [7]. Jawish et al., in another study, also reported a case of a seven year old boy [8]. The cause of cruciate ganglion cysts remains unclear and the literature presents diverging views as to its origin and inception. In this study, no antecedent knee trauma was found in any of the 31 patients. However, tissues of synovial membrane, collagen, and fascia were discovered by histological examination. Therefore, it could be hypothesized that some repeated minor knee trauma contributed to the development of the cyst. The fact that the cysts occurred predominantly in males (male : female ratio, 21:10) also supports this conclusion as females are traditionally considered to be less likely to suffer trauma and sporting injuries, a fact also supported by meta-analyses on the subject [9]. The position of cysts mainly behind the ACL suggested the possible cause could be a result of mechanical force and microtrauma associated with repetitive knee motion.

In this study, the clinical manifestations of ganglion cysts of the cruciate ligaments are varied and often non-specific. Literature review indicated that intra-articular ganglions of the knee could be both symptomatic and asymptomatic [1,4-6,10-13]. Most patients in the study presented with pain and described it mainly around joint line, accompanied with some restriction in flexion or extension because of the worsening pain. The incidence, severity, and duration of pain seem to vary depending on size and location of the cyst. Cysts located mainly anterior to cruciate ligaments tended to limit extension of the knee, whereas those located predominately posterior to the cruciate ligaments tended to limit flexion. It could be speculated that the changes in the length and torsion of the cruciate ligaments, due to knee motion, might result in traction or compression on the cysts that may stimulate the nerve endings on adjacent synovium and result in pain and abnormal sensation.

Differential diagnosis of cruciate ganglion cysts can be excluded safely by relying on the typical MR findings seen in this condition. Before the advent of MRI, these anterior cruciate ligament ganglia were identified only at open surgery or arthroscopy. MR imaging is a valuable tool in diagnosing cysts, especially when the patient presents without any specific history of trauma. In MR images, ganglion cysts demonstrate fluid characteristics with low signal intensity on T1-weighted images and increased signal on T2-weighted images. They are well-delineated structures, appearing as lobulated or multilobulated structures, and are easily distinguishable from Baker cysts or menisci cysts on the T2-weighted images. Usually located within or surrounding the cruciate ligament, these structures do not extend to the medial and lateral head of the gastrocnemius or are connected with meniscus. For the purpose of this study a relatively low tesla machine of 0.2 T, along with a section thickness of 4.5 mm was used. This might have theoretically decreased the sensitivity of detection of some small lesions for which high filed intensity MR scans may be more sensitive.

With the advent of arthroscopy, the treatment of cruciate ligament cysts becomes simple with a successful outcome. In our patients, no recurrence was found. A review of the literature indicated that arthroscopy with cyst removal or aspirate is recommended and always results in complete resolution without injury to adjacent structures [1,4-6,10-13]. A combined arthroscopic and open approach is considered appropriate when cysts are associated with other intra-articular lesions [14]. In our study, the 11 patients who received arthroscopic operation all had cysts that were clearly observed by a routine anteromedial or anterolateral approach. At arthroscopy, the cysts mainly presented as round- or ellipse-shaped, were associated with cruciate ligaments, and had a clear boundary from adjacent structures.

In our practice, preoperative MRI in diagnosis of ligament cyst aids the surgeon in planning ahead with respect for need for special instruments which in this case was a wide or larger angled arthroscope. In order to protect the cyst wall from damage and prevent exudation of the content from the cyst, a blunt dissection should be carried out using an arthroscopic probe. If the cyst is excessively large, fluid can be aspirated before attempting removal with a shaver. Blood vessels in synovium at the surface of ligament should be preserved and, if necessary, ablated with Radiofrequency ablator in order to avoid hemorrhage. One patient in our study had an acute hematoma in the knee joint that was treated with arthrotomy and subsequent drainage. All 11 patients in our study were symptom-free after complete excision of cyst wall by arthroscopy.

## Conclusion

Ganglion cysts of cruciate ligaments are uncommon disorders with non-specific symptoms. A clinical history of pain with the extremes of extension and flexion may be due to several reasons and it is necessary that ganglion cyst is considered one of them. MR imaging is important for confirming the cyst lesions and provides useful information for preoperative planning before the arthroscopic procedure is performed. It also helps to exclude any other associated intraarticular lesion. The MR image and arthroscopic correlation of ligament cysts as demonstrated in our study reinforces the value of preoperative MR Image in patients with chronic knee pain with unclear etiology. Arthroscopic debridement of ganglion cyst offers excellent outcome without recurrence.

## Competing interests

The authors declare that they have no competing interests.

## Authors’ contributions

YT Mao and QR Dong contributed in the conception and design of the study, in the acquisition of data, analysis and interpretation of data, drafting the manuscript. QR Dong also contributed in the final approval of the version to be submitted. Y Wang contributed in the acquisition and analysis of MR image. All authors read and approved the final manuscript.

## Pre-publication history

The pre-publication history for this paper can be accessed here:

http://www.biomedcentral.com/1471-2474/13/137/prepub
